# Adenosine deaminase 2 activity negatively correlates with age during childhood

**DOI:** 10.1186/s12969-020-00446-5

**Published:** 2020-07-10

**Authors:** Sarah M. Bowers, Kristen M. Gibson, David A. Cabral, Kelly L. Brown

**Affiliations:** 1 British Columbia Children’s Hospital Research Institute, Rm A4-145, 950 West 28th Ave, Vancouver, BC V5Z 4H4 Canada; 2 Centre for Blood Research, The University of British Columbia, Vancouver, BC Canada; 3 Department of Medical Genetics, The University of British Columbia, Vancouver, BC Canada; 4 Department of Pediatrics, The University of British Columbia, Vancouver, BC Canada; 5 Division of Rheumatology, British Columbia Children’s Hospital, Vancouver, BC Canada

**Keywords:** Adenosine deaminase 2, Adenosine, Pediatrics, Inflammation

## Abstract

**Background:**

Human adenosine deaminase 2 (ADA2) is an extracellular enzyme that negatively regulates adenosine-mediated cell signaling by converting adenosine to inosine. Altered ADA2 enzyme activity has been associated with some viral infections and rheumatic diseases. The potential utility of ADA2 as a biomarker is, however, limited by the absence of established ranges of ADA2 concentration and enzyme activity in the healthy population. It is known that ADA2 enzyme activity is lower in adults, but when (and why) this decline happens is not known. The purpose of this study was to establish normative ranges of ADA2 enzyme activity and protein concentration in the healthy pediatric population.

**Methods:**

We modified a commercially available ADA2 enzyme activity assay to enable higher throughput analysis of fresh, frozen and hemolyzed blood samples. With this assay and ADA2 protein immunoblotting, we analyzed ADA2 enzyme activity and protein concentration in blood plasma from a cohort of children and adolescents (*n* = 94) aged 5 months to 18 years. One-way ANOVA with subsequent Tukey multiple comparison test was used to analyze group differences. Reference intervals were generated using the central 95% of the population (2–97.5 percentiles).

**Results:**

ADA2 enzyme activity was consistent in fresh, frozen, and hemolyzed sera and plasma as measured by our modified assay. Analysis of plasma samples from the healthy pediatric cohort revealed that ADA2 enzyme activity is significantly lower in older children than in younger children (*p* < 0.0001). In contrast, there was no significant correlation between ADA2 protein concentration and either age or ADA2 enzyme activity.

**Conclusion:**

We observed that ADA2 enzyme activity, but not ADA2 protein concentration, negatively correlates with age in a cohort of children and adolescents. Our findings stress the importance of appropriate age-matched controls for assessing ADA2 enzyme activity in the clinical setting.

## Background

Adenosine deaminases (ADA) catalyze the deamination of adenosine and 2′-deoxy-adenosine to inosine and 2′-deoxy-inosine, respectively. Adenosine deaminase 1 (ADA1) and 2 (ADA2) are two ADA isoenzymes in humans. While both ADA1 and ADA2 share the same substrate, they differ in terms of expression, cellular location, and catalytic and biochemical properties [1–5].

ADA1 is a monomeric, primarily intracellular adenosine deaminase [6]. ADA1 deficiency in humans and mice results in severe combined immune deficiency (SCID), a condition characterized by an accumulation of toxic adenosine metabolites and profound lymphocyte apoptosis [7]. Conversely, ADA2 is a homodimeric, extracellular adenosine deaminase. ADA2 has a lower affinity for adenosine (100-fold higher K_m_) than ADA1, and functions optimally in a hypoxic and/or slightly acidic (pH 6.5) environment, for example, at sites of inflammation [8]. While ADA2 exists in most vertebrates and invertebrates, there is no murine *ADA2* ortholog [9]. In humans, loss of ADA2 enzyme activity is associated with Deficiency of ADA2 (DADA2). DADA2 is an autosomal recessive disease characterized by a range of systemic vascular and inflammatory manifestations, stroke, and in some cases, immune deficiency [10–12]. ADA2 activity has also been studied in a number of adult rheumatic conditions and increased adenosine deaminase activity is observed in individuals with rheumatoid arthritis (RA) [13, 14], systemic lupus erythematous (SLE) [15], and Crohn’s disease [16]. ADA2 enzyme activity is also elevated and may be used as a diagnostic tool for tuberculosis [17] and HIV [18] infection, as well as for assessing benign versus malignant tumors [19]. These data suggest a regulatory role for ADA2 in inflammation and a potential to develop ADA2 as a diagnostic or disease activity biomarker for inflammatory diseases.

The implementation of clinical testing for ADA2 is impeded by the absence of (i) normative ranges for ADA2 activity and concentration in adults and children, and (ii) simple, inexpensive, high-throughput ADA2 assays. Assessment of ADA2 enzyme activity in clinical samples is typically done by spectrophotometry [20, 21] or high performance liquid chromatography [10]. While it may not be necessary for diagnostic purposes to differentiate between abnormal ADA2 activity due to alterations in catalytic activity versus the presence of unusually high or low concentrations of ADA2 protein, the distinction is needed to inform disease mechanism and appropriate therapy. Moreover, without standardized, age-specific reference ranges for normal ADA2 activity and protein concentration, testing is limited to the identification of extreme cases in which ADA2 catalytic activity/expression is abolished. The consideration of age is especially important for pediatric cases in light of observations that ADA2 mRNA and ADA2 activity is lower in healthy adults than in healthy children [10, 20] – though when and why this decline happens is not yet understood.

In this study, we adapted a commercially available, ADA2 activity assay to enable fast and inexpensive testing of ADA2 activity in clinical samples. We demonstrate that the assay is compatible with small volumes (10 μL) of fresh, previously frozen and/or hemolyzed blood samples. We then used this enzyme assay and western blot to measure ADA2 activity and concentration in the plasma of healthy children and adolescents under 18 years of age. We report age-specific normative ranges of ADA2 activity and demonstrate that ADA2 activity, but not necessarily ADA2 protein, steadily declines throughout childhood and adolescence.

## Methods

### Participants

Healthy adults and children diagnosed with Deficiency of adenosine deaminase 2 (DADA2) were enrolled in the Pediatric Vasculitis Initiative (PedVas) as described previously [21, 22]. The study protocol was approved by the Children’s and Women’s Research Ethics Board of the University of British Columbia (H12–00894) and the respective ethical committees or IRBs at participating PedVas study sites. One hundred otherwise healthy children and young adults (age range: 3 months – 18 years) followed at British Columbia (BC) Children’s Hospital (BCCH, Vancouver, BC, Canada) for sleep apnea or epilepsy were enrolled to the BCCH BioBank; study protocol approved by the Children’s and Women’s Research Ethics Board of the University of British Columbia (H13–03111). Participants were screened for underlying immune activation by analysis of C-reactive protein (CRP) concentration in plasma (1:2000 dilution, C-reactive protein ELISA kit, ThermoFisher, MA, USA).

### Blood sample collection and processing

Participant sera and/or plasma was obtained following centrifugation (1200 x *g* for 10 min) of whole blood within 30–120 min of blood collection in K_2_EDTA or SST™ tubes (Becton, Dickinson, NJ, USA). Aliquots of sera/plasma were stored at − 80 °C prior to analysis. To test the effect of repeat freeze-thaw on the stability of ADA2, plasma and sera were subject to 1, 2 and 3 cycles of incubation for 10 min on dry ice followed by 30 min in a 37 °C incubator. To test the potential interference of ADA1 released from lysed red blood cells, hemoglobin (HGB) was measured (pocH-100i hematology analyzer, Sysmex, Japan) in whole blood then diluted 1:1 either in PBS (non-hemolyzed) or in deionized water and incubated at room temperature for 20 min, on dry ice for 10 min, and at 37 °C for 30 min (hemolyzed). Samples were centrifuged at 1000 x *g* for 10 min to obtain plasma in which HGB concentration was measured and percent hemolysis calculated as ([plasma HGB]/[whole blood HGB]) × 100. Samples were diluted 1:5 in PBS prior to ADA activity assay.

### Adenosine deaminase (ADA) activity assay

An adenosine deaminase assay (DZ117A, Diazyme Laboratories Inc., CA, USA) was modified and used to measure ADA2 enzyme activity. Samples (10 μL of serum or plasma) were equilibrated at 37 °C with Reagent 1 (R1; 180 μL). An initial absorbance (556 nm) was measured (A1), Reagent 2 (R2; 90 μL) added and the reaction allowed to proceed. Absorbance was recorded every 2 min for 10 min, then once every 10 min for up to 8 h to establish an optimal end point read (A2) at 180 min. To measure ADA2 specific activity, erythro-9-(2-hydroxy-3-nonyl) adenine hydrochloride (EHNA; Millipore Sigma, ON, Canada), an ADA1-specific inhibitor [23] was added prior to addition of R1 (17.54 μM final concentration in the assay). ADA (ADA1 and ADA2) calibrator (30 U/L; Diazyme) and 0.9% saline were used as standards and controls to generate a linear slope of absorbance (A2 - A1) versus ADA activity (U/L) from which the equation of the line was used to calculate total ADA activity (ADA1 and ADA2) or ADA2-specific activity. Where appropriate, experiments used recombinant human (rh) ADA1 and ADA2 proteins (R&D Systems, MN, USA).

### Quantification of ADA2 protein by SDS-PAGE and western blot

All reagents from ThermoFisher, MA, USA unless specified otherwise. Plasma (1:50 dilution in PBS) and rhADA2 (5, 10, 20 ng) were resolved by SDS-PAGE (4–12% NuPage gels), transferred to an Immobilon-P 0.45 μM PVDF membrane (Millipore Sigma, MA, USA), blocked (5% skim milk powder in PBS) and probed with anti-CECR1 (anti-ADA2) polyclonal antibody (1:1000 dilution, PA5–30635) followed by an HRP-conjugated goat anti-rabbit IgG (H + L) antibody (1:20,000 dilution, A16104). Membranes were incubated with SuperSignal™ West Pico PLUS chemiluminescent substrate, exposed to autoradiography film (Diamed, ON, CAN) and imaged with AlphaImager 2200 (Alpha Innotech, CA, USA). Protein bands were compared to prestained protein ladder (PageRulerPlus #26619) for relative molecular weight, and densitometry analysis was performed using ImageJ 1.52a software [24]. ADA2 concentration in samples was obtained from a linear standard curve of rhADA2 protein(ng) vs. band density.

### Statistical analyses

Statistical tests were performed using GraphPad Prism version 7.0c for Mac OS X (GraphPad Software, La Jolla California, USA). Linear regression and correlation were used for scatter plots. One-way ANOVA with subsequent Tukey multiple comparison test was used to analyze group differences. Outliers were determined by the ROUT test, where Q = 1%, and excluded from further statistical analyses. For all analyses a confidence interval of 95% was used. Reference intervals were generated using the central 95% of the population (2–97.5 percentiles) as recommended [25].

## Results

### ADA2 enzyme assay resolution is improved and linearity retained with extended reaction time

To simplify ADA2 enzyme activity assessment, we modified a common assay to enable end-point absorbance (556 nm, Supplementary Figure 1, Additional file 1) readings of samples in a 96-well microplate. We determined the optimal end-point read time that would both enhance detection of relatively low (in adults) and high (in children) ADA2 activity while maintaining assay linearity. To do this, we performed the assay over a time course from 10 min (as per manufacturer’s instructions) to 8 h (Supplementary Figure 2A – 2B, Additional file 1) using sera and plasma from healthy individuals and from children with abrogated ADA2 enzyme activity due to DADA2 [21]. At the earliest time point (10 min; Supplementary Figure 2A, Additional file 1), ADA2 activity in plasma from otherwise healthy children was significantly higher than in samples from children with DADA2 (*p* < 0.0001, *n* = 5) and healthy adults (*p* < 0.0001, *n* = 5). However, ADA2 activity in healthy adults was not distinguishable from ADA2 activity in children with DADA2 at this time point (*p* = 0.8316, *n* = 5). By 3 h, a significant difference in ADA2 activity in healthy adults and children with DADA2 was observed (Supplementary Figure 2B, Additional file 1). To assess assay linearity over this time period, the supplied calibrator was serially diluted and a change in absorbance over time was measured (Supplementary Figure 2C, Additional file 1). Data were fit with the linear eq. Y = 0.07639X + 0.3018, where Y is the change in absorbance and X is ADA2 activity in U/L (*n* = 3, R^2^ = 0.9952). Our results show that the assay remained linear over the 3 h period (Supplementary Figure 2D, Additional file 1) and is capable of detecting 1.0–38.0 U/L of ADA2 activity. The coefficient of variation (CV) was < 0.075 for each time-point (*n* = 3), indicating highly reproducible results and negligible inter-assay variance in blood samples from healthy adults and children.

### ADA2 enzyme assay is compatible with hemolyzed and stored blood samples

**Fig. 1:**
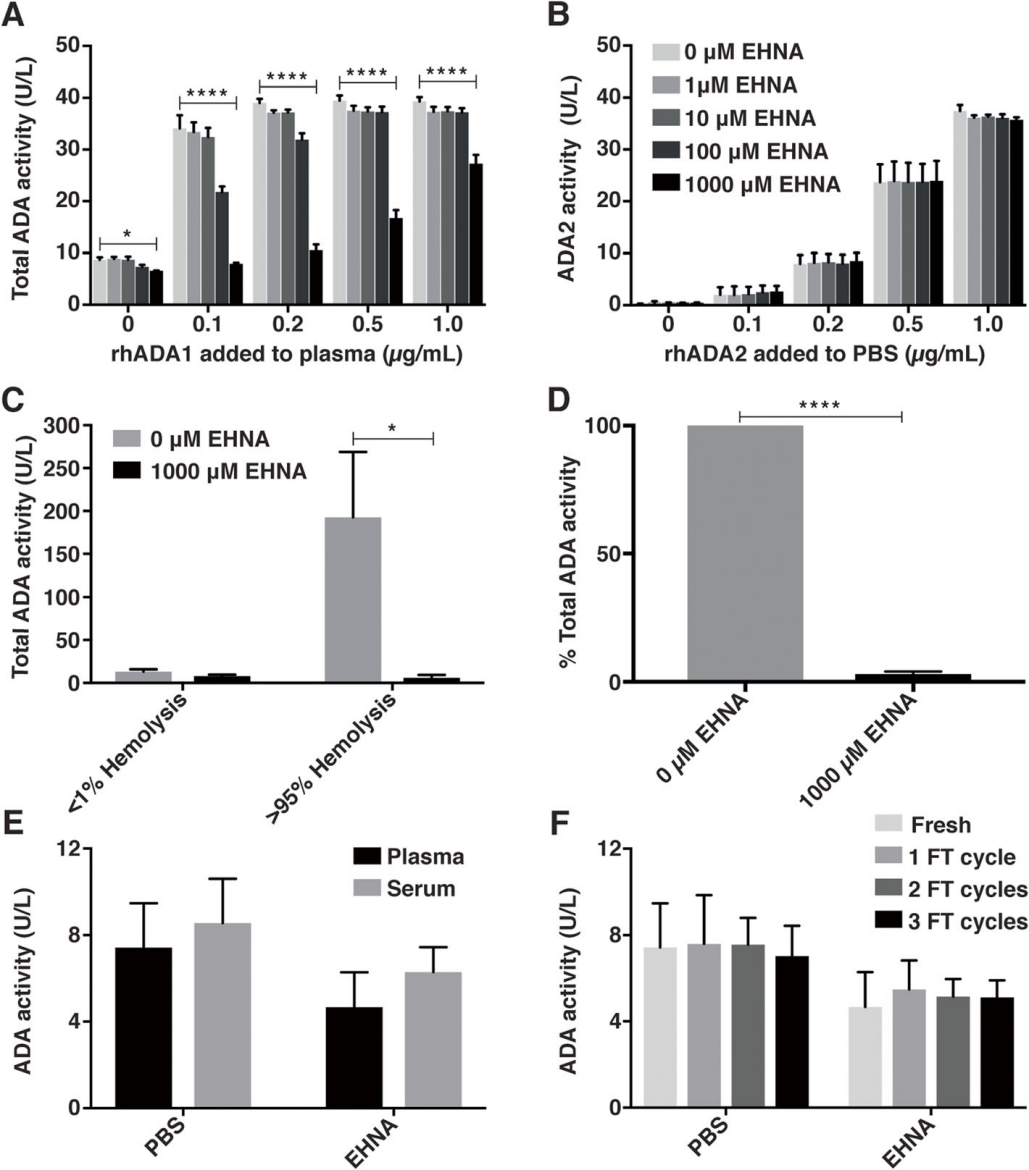
Assay optimization for analysis of ADA2 activity in plasma and serum **a** Total ADA activity (ADA1 and ADA2) (y-axis: Total ADA activity (U/L)) in serial dilutions of rhADA1 in plasma (x-axis: rhADA1 added to plasma (0–1 μg/mL), *n* = 3) and **b** ADA2 activity (y-axis: ADA2 activity (U/L)) in serial dilutions of rhADA2 in PBS (x-axis: rhADA2 added to PBS (0–1 μg/mL), *n* = 3) in the presence of 5 μL of increasing concentrations (0, 1, 10, 100, 1000 μM) of EHNA per 10 μL of sample in an assay volume of 285 μL. **c** Total ADA activity (y-axis: ADA (U/L)) in plasma samples from non-hemolyzed whole blood (x-axis: < 1% hemolysis, *n* = 3) and hemolyzed whole blood (x-axis: > 95% hemolysis, *n* = 3) in the absence (grey bars) and presence (black bars) of 5 μL of 1000 μM EHNA. **d** Percent of total ADA activity (y-axis: % Total ADA activity, *n* = 3) in plasma samples from hemolyzed whole blood in the absence (x-axis: 0 EHNA) and presence of EHNA (x-axis: 5 μL of 1000 μM EHNA). Significance only shown for the highest concentration of EHNA. **e–f** ADA enzyme activity (y-axis: ADA activity (U/L)) measured in the absence (x-axis: PBS: total ADA1 and ADA2 activity) and presence of ADA1 inhibitor, EHNA (x-axis: EHNA: only ADA2 activity) in **e** fresh plasma and serum (*n* = 3 adults) and **f** plasma subjected to freeze thaw (FT) cycles (*n* = 3 adults). Bars show mean + SD. * *p* < 0.05, ** *p* < 0.01, *** *p* < 0.001, **** *p* < 0.0001

To guard against false positive ADA2 enzyme activity due to ADA1 that is either naturally present at trace amounts in serum and plasma or is inadvertently released from hemolyzed cells during blood collection and processing, a commonly used specific inhibitor of ADA1, EHNA [23], is included in the assay protocol. To determine an optimal concentration of EHNA that blocks ADA1 in the assay, increasing concentrations of recombinant human (rh) ADA1 (0.1–1 μg/mL) in plasma (pooled from *n* = 6 healthy adult donors) were measured in the absence (total activity from ADA1 and ADA2) and presence of increasing concentrations of EHNA (addition of 5 μL of 1–1000 μM EHNA). In the absence of rhADA1, EHNA had a small inhibitory effect on ADA activity in plasma, which concurs with reports of trace amounts of endogenous, extracellular ADA1. The addition of low concentrations of rhADA1 (0.2 μg/mL) was sufficient to saturate the assay (Fig. 1a) and emphasizes the importance of blocking ADA1 for the analysis of clinical samples. Our results show that the highest tested concentration of EHNA (5 μL of 1000 μM; 17.54 μM final concentration in the assay) reduced ADA activity in plasma samples spiked with low concentrations of ADA1 (0.1 μg/mL) to levels that are comparable (*p* = 0.2774, *n* = 3) to endogenous ADA2 activity in plasma alone (plasma + 5 μL of 1000 μM EHNA). To confirm that concentrations of EHNA do not block ADA2 activity, increasing concentrations of rhADA2 (0.1–1 μg/ml in PBS) were assayed in the absence and presence of increasing concentrations of EHNA. ADA activity increased linearly with rhADA2 concentration in the absence of EHNA, and was not altered (*p* = 0.6863, *n* = 3) by the presence of even the highest concentration of EHNA (Fig. 1b).

To evaluate the ability of EHNA to effectively inhibit physiologically relevant plasma concentrations of ADA1, particularly ADA1 released from hemolyzed blood cells, ADA activity was measured in plasma obtained from non-hemolyzed and hemolyzed blood generated ex vivo (Fig. 1c – d). Using measures of hemoglobin (HGB) as an indicator of cell lysis, we estimate less than 1% cell lysis during standard processing of blood (*n* = 3; see Methods for calculation). Higher ADA activity was observed (*p* = 0.051) in plasma from hemolyzed blood (192.7 +/− 76.2 U/L, *n* = 3) compared to plasma obtained from standard blood processing (13.1 +/− 2.9 U/L, *n* = 3). EHNA (5 μL of 1000 μM) significantly reduced ADA activity in these hemolyzed samples by ~ 97.0 +/− 1.0% (*p* < 0.0001, *n* = 3, Fig. 1d), which was comparable to ADA activity in plasma obtained by standard blood processing (*p* = 0.9978, *n* = 3, Fig. 1c).

As blood draw volumes in young patients are often limited, an assay that is compatible with samples (e.g. sera or plasma) that may be leftover from clinical testing (either fresh or frozen) is advantageous. To determine if ADA2 activity is comparable in plasma and sera, ADA2 activity in fresh serum and plasma was measured (Fig. 1e). No significant difference in ADA2 activity was observed between fresh sera and plasma (*p* =  0.0774, *n* = 3), suggesting that either could be used to measure ADA2 enzyme activity. To determine the necessity for fresh samples, ADA activity in plasma was measured before and after samples were subjected to repeat freeze-thaw cycles (Fig. 1f). Compared to fresh plasma, no significant reduction in total ADA2 activity (*p* = 0.9114, 0.9793, 0.9838 for 1, 2, and 3 freeze-thaw cycles, respectively; *n* = 3) was observed, demonstrating that previously frozen plasma samples are also suitable for ADA2 activity analysis. Our results also demonstrate that ADA1 is resistant to repeat freezing and thawing, further emphasizing the importance of ADA1 inhibition in the assay.

### ADA2 enzyme activity in plasma negatively correlates with age through adolescence

**Fig. 2:**
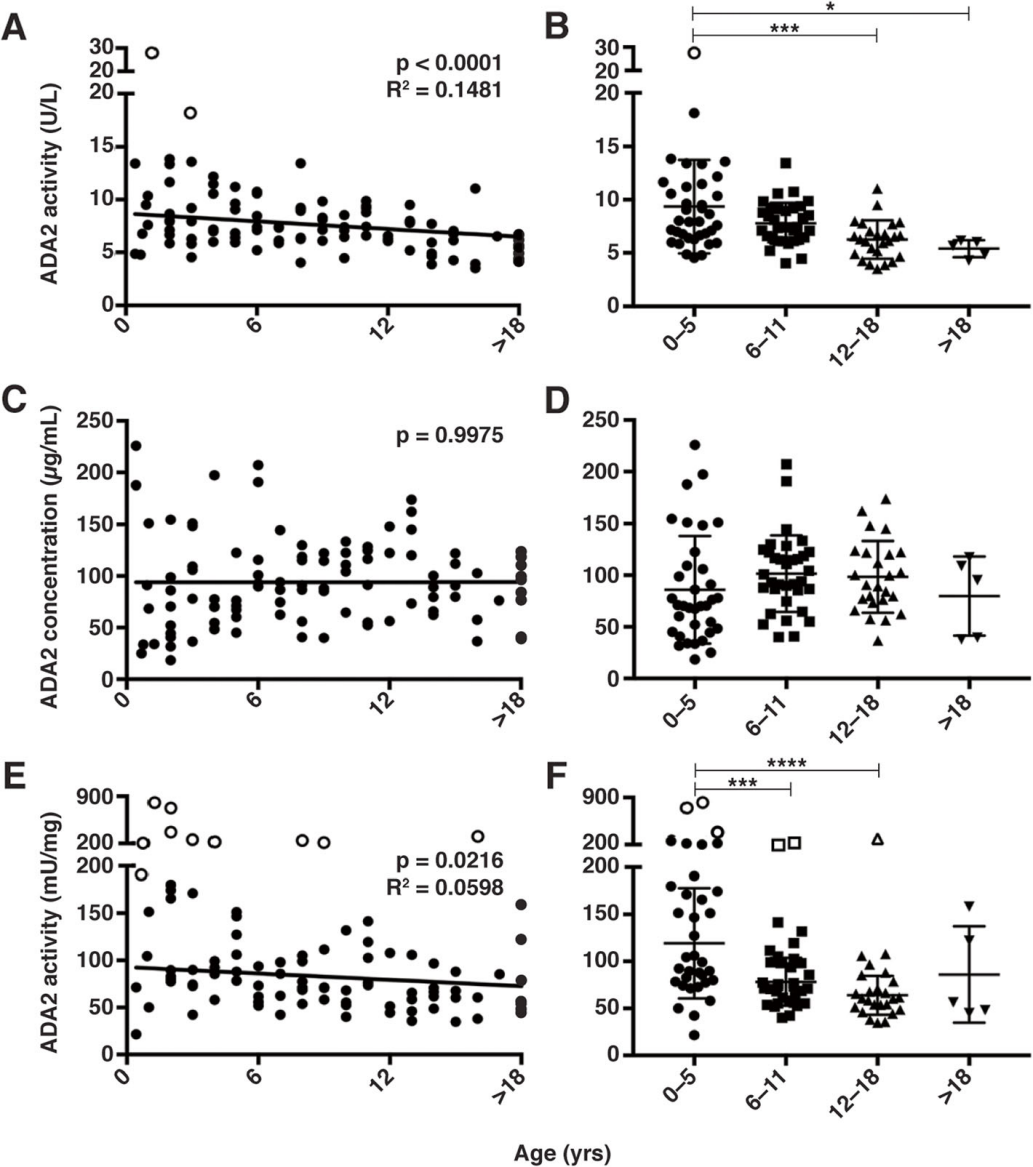
Plasma ADA2 enzyme activity and concentration in a healthy pediatric cohort. Plasma samples from otherwise healthy children and adolescents (x-axis, unless otherwise noted) aged 0–2 yrs (*n* = 16), 3–5 yrs (*n* = 19), 6–8 yrs (*n* = 18), 9–11 yrs (*n* = 16), 12–14 yrs (*n* = 13), 15–18 yrs (*n* = 12) and healthy adults (> 18 yrs, *n* = 5) were analyzed. **a–b** ADA2 enzyme activity (y-axis: ADA2 activity (U/L)). **c–d** ADA2 concentration (y-axis: ADA2 concentration (μg/mL)). **e–f** ADA2 activity normalized to ADA2 concentration (y-axis: ADA2 activity (mU/mg). Trend lines show linear regression of data. Outliers are denoted by open shapes and were excluded from statistical analyses. * *p* < 0.05, ** *p* < 0.01, *** *p* < 0.001, **** *p* < 0.0001

It has been previously shown that ADA2 activity in blood samples from adults is lower than in children [10, 20] but it is not known at what age ADA2 activity begins to decline. To evaluate changes in ADA2 activity that correlate with age, ADA2 activity was measured in plasma obtained from 100 healthy children and adolescents, and from 5 adults (> 18 yrs) (Fig. 2a). Concentrations of C-reactive protein (CRP), a known biomarker of inflammation [26], were within the normal range (< 5 μg/ml [27]) for 94/100 healthy participants aged 0–2 yrs (*n* = 16; youngest being 5 months of age), 3–5 yrs (*n* = 19), 6–8 yrs (*n* = 18), 9–11 yrs (*n* = 16), 12–14 yrs (*n* = 13), 15–18 yrs (*n* = 12). Samples from 6 individuals with elevated CRP (suggestive of underlying inflammatory processes) were excluded from further analysis (Supplementary Figure 3, Additional file 1). Analysis of samples from the healthy pediatric cohort (*n* = 94) revealed a negative correlation between ADA2 activity and age (*p* < 0.0001, Fig. 2a). A comparative analysis of results between age groups representing infancy (0–5 yrs), early adolescence (6–11 yrs), adolescence (12–18 yrs), and adulthood (> 18 yrs), demonstrated that ADA2 activity was significantly lower in adolescents and adults in comparison to infants (*p* = 0.0005 and 0.0173, respectively, Fig. 2b). Age-specific reference intervals for ADA2 activity were generated with the central 95% range (2.5–97.5 percentiles) [25] to be: 4.7–14.7 (ages 0–5 yrs), 4.3–11.4 (ages 6–11 yrs), 3.6–10.6 (ages 12–18), and 4.3–6.2  (> 18 yrs) (U/L) (Table 1).

**Table 1:** Reference intervals (central 95%) of ADA2 activity and protein concentration in healthy children and adolescents

Age Range (yrs)	Lower Limit (2.5 percentile)	Upper Limit (97.5 percentile)
**ADA2 enzyme activity (U/L)**		
0–5	4.7	14.7
6–11	4.3	11.4
12–18	3.6	10.6
> 18	4.3	6.2
**ADA2 protein concentration (μg/mL)**		
0–5	23.8	203.1
6–11	40.5	194.9
12–18	42.6	170.5
> 18	37.9	117.3
**Normalized ADA2 enzyme activity (mU/mg)**		
0–5	35.0	232.5
6–11	41.6	135.2
12–18	35.1	107.4
> 18	45.0	158.4

### ADA2 concentration in plasma does not decline with age

**Fig. 3:**
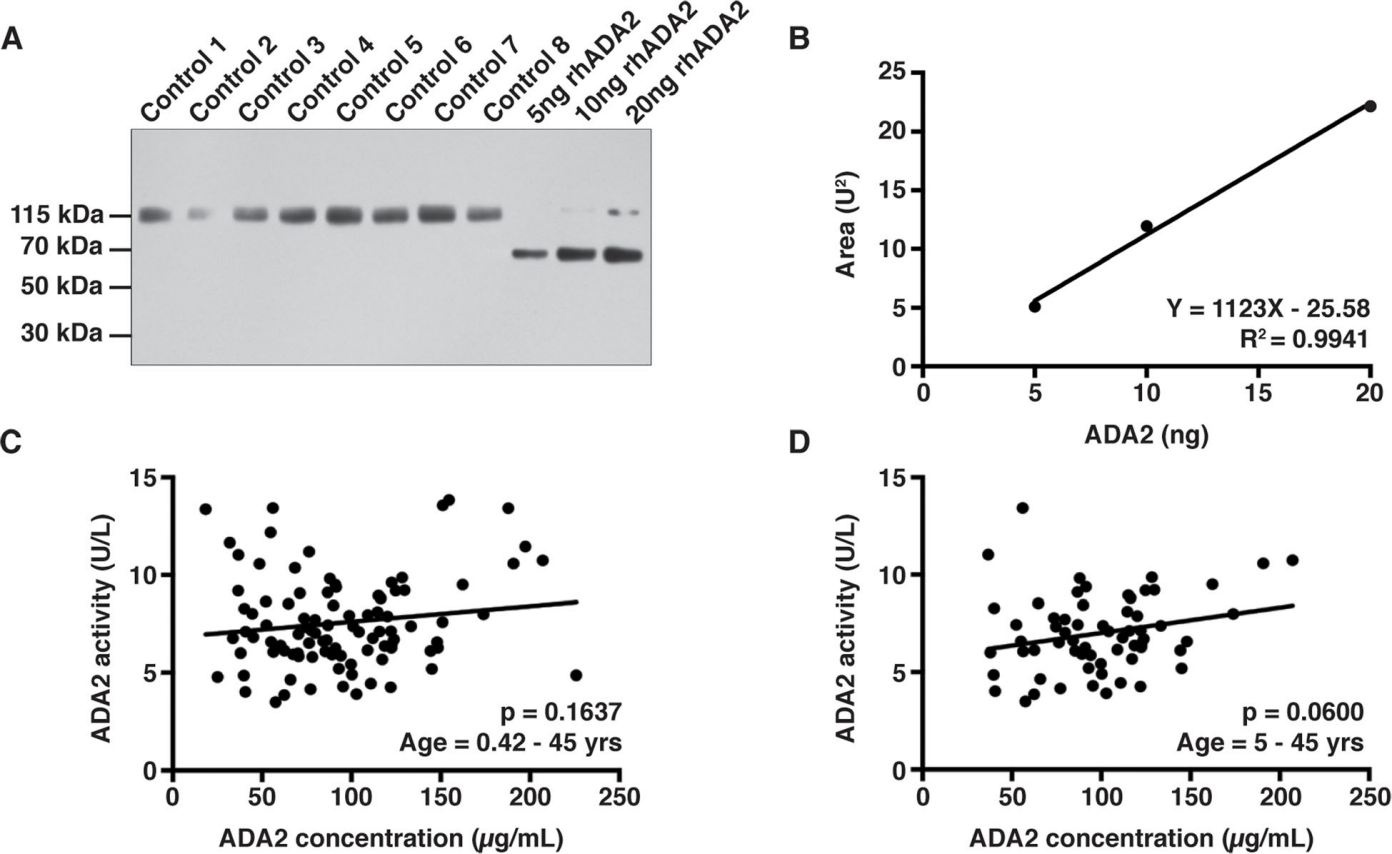
Quantitation of plasma ADA2 protein. **a** Representative western blot of ADA2 in plasma (Control 1–8) and recombinant human (rh) ADA2 (5, 10, 20 ng rhADA2). **b** Representative standard curve of rhADA2 (y-axis: Area (square units); x-axis: ADA2 (ng)). **c** ADA2 activity (y-axis: ADA2 activity (U/L)) compared to ADA2 protein concentration (x-axis: ADA2 concentration (μg/mL)) for each adult and pediatric healthy control (*n* = 99, age = 5 months–45 yrs) and **d** for controls > 5 yrs of age (*n* = 64). Outliers are denoted by open circles and were excluded from statistical analyses

To determine if the decline in ADA2 activity observed in plasma with respect to age was reflected in lower concentrations of ADA2 protein, ADA2 in participant plasma was resolved by non-reducing SDS-PAGE, detected by immunoblotting, and quantitated by densitometry analysis. A representative immunoblot shows dimeric ADA2 in control plasma and both dimeric and monomeric rhADA2 (Fig. 3a). ADA2 concentrations were calculated from a standard curve generated with known amounts of rhADA2 on each gel (Fig. 3b). Unlike ADA2 activity, no correlation between ADA2 concentration and age was observed (Fig. 2c). In addition, ADA2 concentration did not correlate with activity (Fig. 3c). However, a trending correlation was observed between ADA2 concentration and activity (Fig. 3d) when data from children 0–5 yrs. of age was excluded due to high variability in ADA2 concentration within this group (Fig. 2d). Across all age groups, ADA2 enzyme activity normalized to concentration was significantly correlated with age (*p* = 0.0216, Fig. 2e). Specifically, normalized ADA2 activity was decreased in age groups representing early adolescence and adolescence in comparison to infancy (*p* = 0.0006 and < 0.0001, respectively, Fig. 2f).

## Discussion

In the current study, we optimized a commercially available ADA1/2 enzyme activity assay for specific assessment of ADA2 enzyme activity in blood samples using a simple microplate procedure with an optimal end-point absorbance read at 3 h. We have shown that our ADA2 enzyme activity assay can detect 1.0–38.0 U/L of ADA2 activity in 10 μL of fresh, frozen, and hemolyzed sera and/or plasma. We demonstrate that ADA2 enzyme activity is negatively correlated with age in childhood, and does not correlate with a significant decline in ADA2 protein concentration. Finally, we use these data to calculate normative ranges of ADA2 activity relative to age in healthy children and adolescents.

The assay described in this study offers an accessible, cost- and time-effective method for ADA2 enzyme activity testing. In the absence of a plate reader enabled for kinetic measurements, an initial (A1) and final (A2) absorbance reading on a standard plate reader are sufficient to determine ADA2 activity. However, care must be taken to ensure that absorbance does not reach saturation, as saturated samples will deviate from the linearity of the assay. In the event that the final absorbance reading of a sample approaches the upper limit of detection, repeat analysis of an appropriate dilution of the sample in PBS will ensure values remain within the linear range. The assay is suitable for both sera and plasma, as well as either fresh or frozen samples. The addition of EHNA (17.54 μM final concentration in the assay) allows for specific quantitation of ADA2 enzyme activity, even in hemolyzed samples. It remains possible that other factors released from hemolyzed cells may compromise the integrity of the sample and/or contribute to assay interference.

While attempts have been made to normalize ADA2 activity to total serum proteins [28] normalization of ADA2 activity to ADA2 protein concentration has not, to our knowledge, been reported previously. Normalizing ADA2 activity to ADA2 concentration reduced variability between individuals. Since not all clinical sites will have access to techniques that allow for ADA2 protein quantitation in clinical samples, we have also reported ranges for absolute ADA2 activity with respect to age in the healthy population. While the assay is compatible with both sera and plasma, these reported ranges are for plasma samples with additional testing required on sera. These reference values will enable analysis on a case-by-case basis of ADA2 activity for diagnosis or disease monitoring without needing to acquire age-matched disease or healthy controls with each clinical sample. Our data suggests that caution should be taken when interpreting reportedly low ADA2 activity (in the absence of total ADA2 protein) in children as activity will naturally decline with age.

Lack of a definitive correlation between ADA2 enzyme activity and ADA2 protein concentration in plasma is a somewhat unexpected result from our study. It may suggest regulatory mechanisms on ADA2 activity in vivo other than regulation of protein expression and secretion. Mechanisms may include conformational changes (dimerization) of ADA2, cofactor availability, and/or post-translational modifications. An increased rate of dimer dissociation has shown to decrease, but not completely abolish enzyme activity [3]. However, our data demonstrates that across all age groups, including adults, quantified protein was in the dimeric form, as seen in the representative immunoblot. Detection of the dimeric form in plasma agrees with the previous literature which states that dimerization is required for ADA2 secretion from the cell [29]. Alternatively, a decrease in available cofactor, primarily zinc (Zn), may be responsible for decreased enzyme activity of ADA2 in vivo. This seems unlikely however given that ADA2, unlike ADA1, does not undergo a substantial conformational change upon binding to Zn [3]. Furthermore, a decrease in plasma Zn has not been reported in early adolescence, although it is reduced in the elderly [30, 31]. Finally, changes in enzyme activity may be due to conformational changes triggered by post-translational modifications. Phosphorylation is a common post-translational modification that may be involved in the regulation of up to 30% of mammalian proteins and is well described for the regulation of signaling molecules, including enzymes [32]. Phosphoramides (histidine, arginine, lysine) are much less stable than the more common phosphomonoesters (serine, threonine, tyrosine) and may be difficult to detect unless isolation and analyses are done in the absence of acidic conditions [33].

## Conclusion

In summary, we have optimized an ADA2 enzyme activity assay for clinical utility and have demonstrated its use with biological samples (sera/plasma). To further the utilization of ADA2 enzyme activity in the clinical or research setting, we have generated normalized ADA2 activity ranges with respect to age to account for the natural decline in ADA2 activity that we demonstrate will occur from infancy to adulthood. Increased testing of ADA2 enzyme activity over a broad range of rheumatic conditions may elucidate ADA2 as a biomarker of disease activity and broaden our understating of ADA2 in inflammation.

## Supplementary information

**Additional file 1: Supplementary figure 1.** Schematic overview of the ADA2 activity assay; **Supplementary figure 2.** Time optimization of ADA2 assay; **Supplementary figure 3.** Plasma CRP concentration in the healthy pediatric cohort.

## Data Availability

All data generated or analysed during this study are included in this published article (and its supplementary information files).

## Abbreviations

ADA: Adenosine deaminase
ADA1: Adenosine deaminase 1
ADA2: Adenosine deaminase 2
CRP: C-reactive protein
CV: Coefficient of variation
DADA2: Deficiency of ADA2
EHNA: Erythro-9-(2-hydroxy-3-nonyl) adenine hydrochloride
HGB: Hemoglobin
PedVas: Pediatric Vasculitis Initiative
RA: Rheumatoid arthritis
SCID: Severe combined immune deficiency
SLE: Systemic lupus erythematous
Zn: Zinc
