# Supplementary material for: Adenosine deaminase 2 activity negatively correlates with age during childhood
Source: Pediatr Rheumatol Online J. 2020 Jul 10;18:54. doi: 10.1186/s12969-020-00446-5 (PMC7350767; doi:10.1186/s12969-020-00446-5)

Supplementary Figure 1. Schematic overview of the ADA2 activity assay

One unit of adenosine deaminase (ADA) activity (U/L) was defined as the amount of ADA (inclusive of ADA1 and ADA2) that generates one µmole of inosine from adenosine per minute at 37°C. Specific ADA2 activity was taken to be the ADA activity of serum/plasma measured in the presence of 5 µL of 1000 µM EHNA, an ADA1 specific inhibitor (final concentration of 17.54 µM EHNA in the total assay volume of 285 µL).

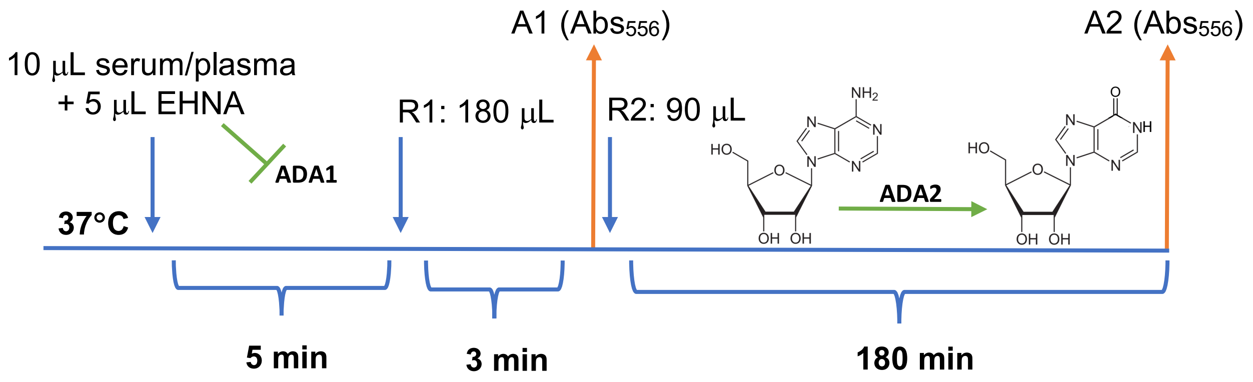

Supplementary Figure 2. Time optimization of ADA2 Assay (A) ADA2 activity (y-axis: ADA2 activity (U/L)) in blood sera or plasma obtained from DADA2 patients (x-axis: DADA2, n = 5), healthy children (x-axis: Pediatric Controls, n = 5), and healthy adults (x-axis: Adult Controls, n = 5) calculated after 10 minutes and **(B)** 3 hours. Horizontal bars show mean ADA2 activity +/- standard deviation (SD). **(C)** Absorbance (y-axis: Abs_{556nm}) measured in serial dilutions of calibrator over 3 hours (x-axis: Time (min)) **(D)** Change in absorbance (y-axis; ΔAbs_{556}) of serial dilutions of calibrator (x-axis: Calibrator (U/L), n = 3) after 3 hours. Points show mean change in absorbance +/- SD and trendline shows linear regression of data. * $p < 0.05$, ** $p < 0.01$, *** $p < 0.001$, **** $p < 0.0001$.

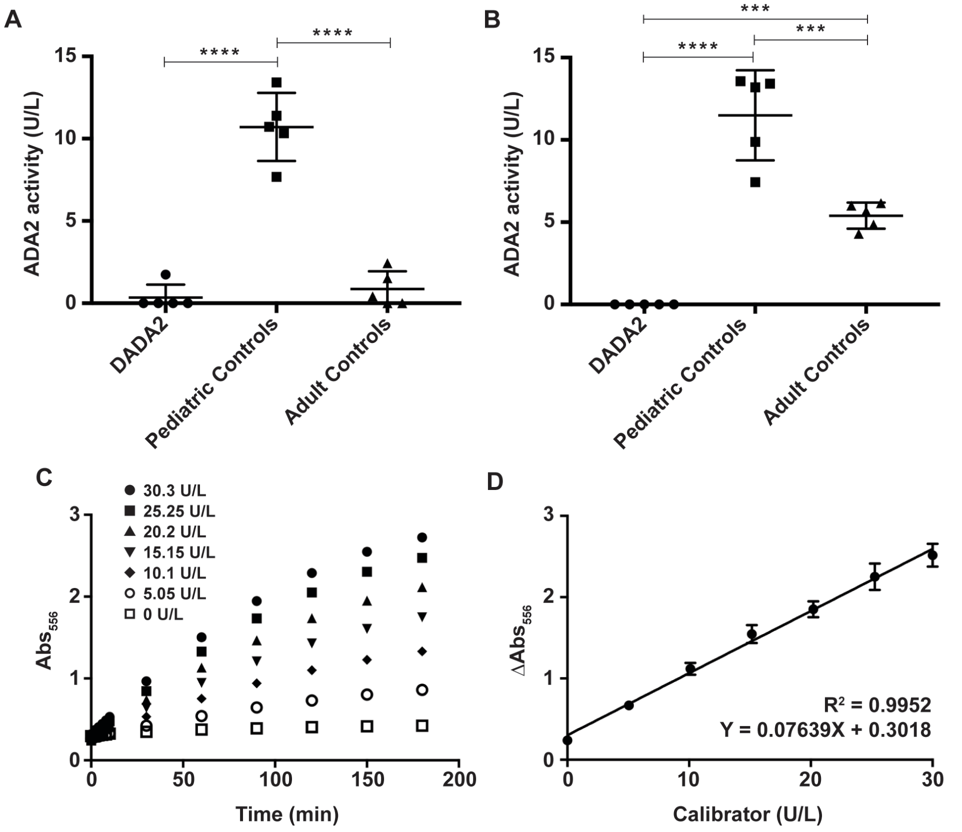

Supplementary Figure 3. Plasma CRP concentration in the healthy pediatric cohort. C-reactive protein (y-axis: CRP µg/ml) quantitated by ELISA in plasma (diluted 1:2000) from otherwise healthy children ages 5 months – 18 yrs (x-axis, n = 94). Dotted line denotes upper limit of normal CRP concentration in healthy children (5 µg/mL).

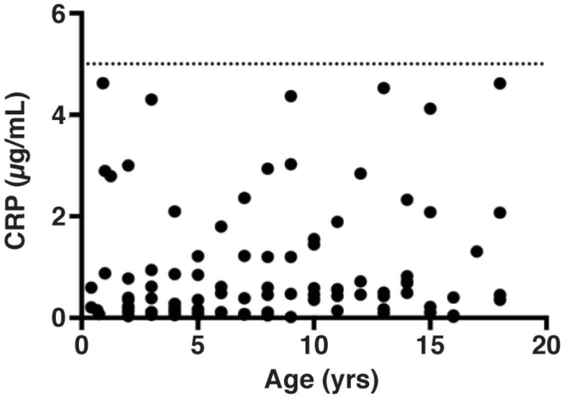

Supplement: Supplementary file 1 — Additional file 1: Supplementary figure 1. Schematic overview of the ADA2 activity assay; Supplementary figure 2. Time optimization of ADA2 assay; Supplementary figure 3. Plasma CRP concentration in the healthy pediatric cohort. [file 12969_2020_446_MOESM1_ESM.pdf]
